# Supplementary material for: Community- and genome-based evidence for a shaping influence of redox potential on bacterial protein evolution
Source: mSystems. 2023 Jun 8;8(3):e00014-23. doi: 10.1128/msystems.00014-23 (PMC10308962; doi:10.1128/msystems.00014-23)
Supplement: FIG S1 — ZC–Eh7 scatterplots and linear regressions for all data sets. Subtitles indicate the number of samples, Pearson correlation coefficient, and slope of the linear regression ± the margin of error for the 95% confidence interval. Regression lines are solid if the slope is > 0.01/V or < −0.01/V, or dashed otherwise. [file msystems.00014-23-s0001.pdf]

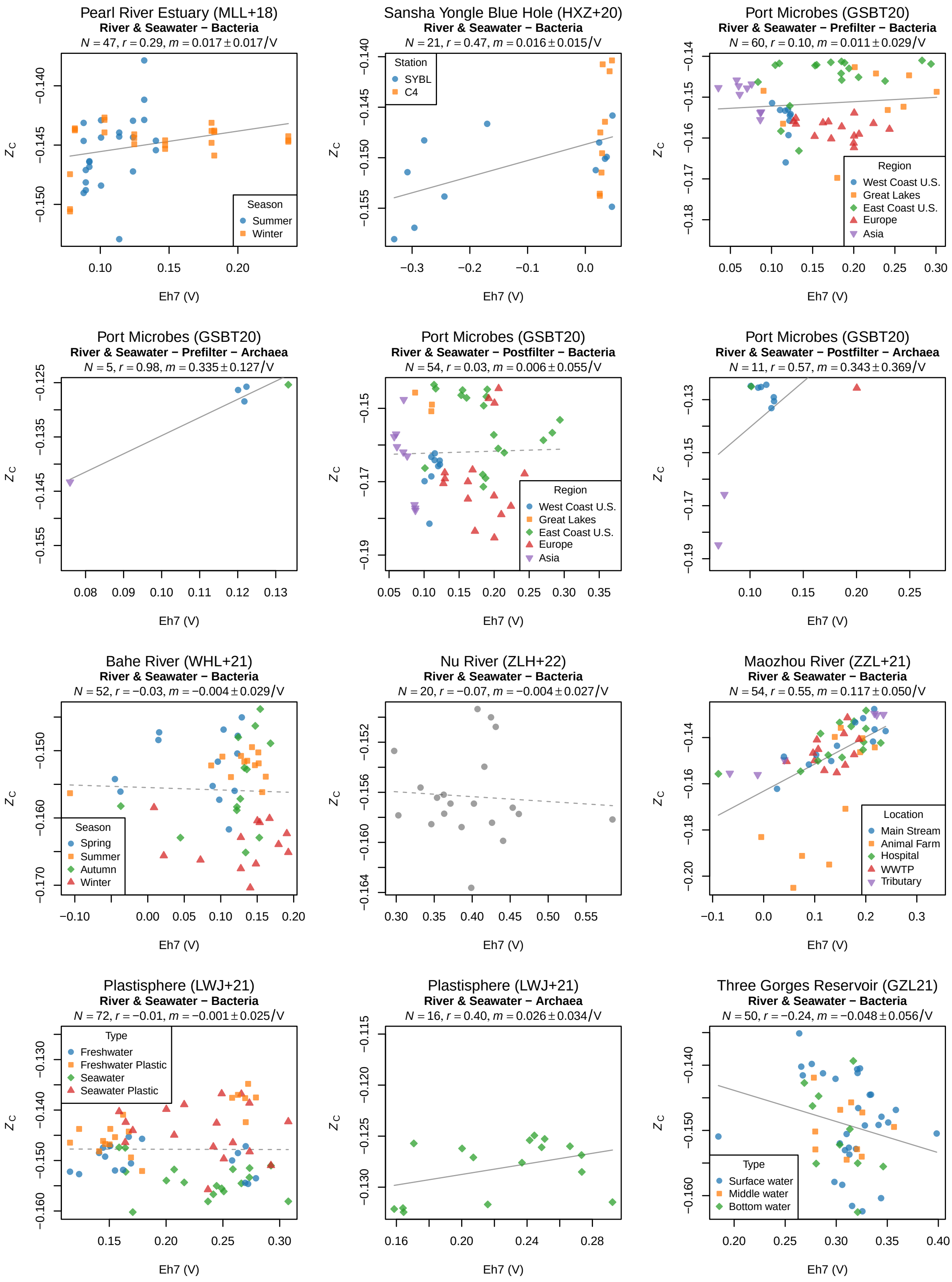

Figure S1. SYBL – Sansha Yongle Blue Hole; C4 – ocean site; WWTP – wastewater treatment plant.

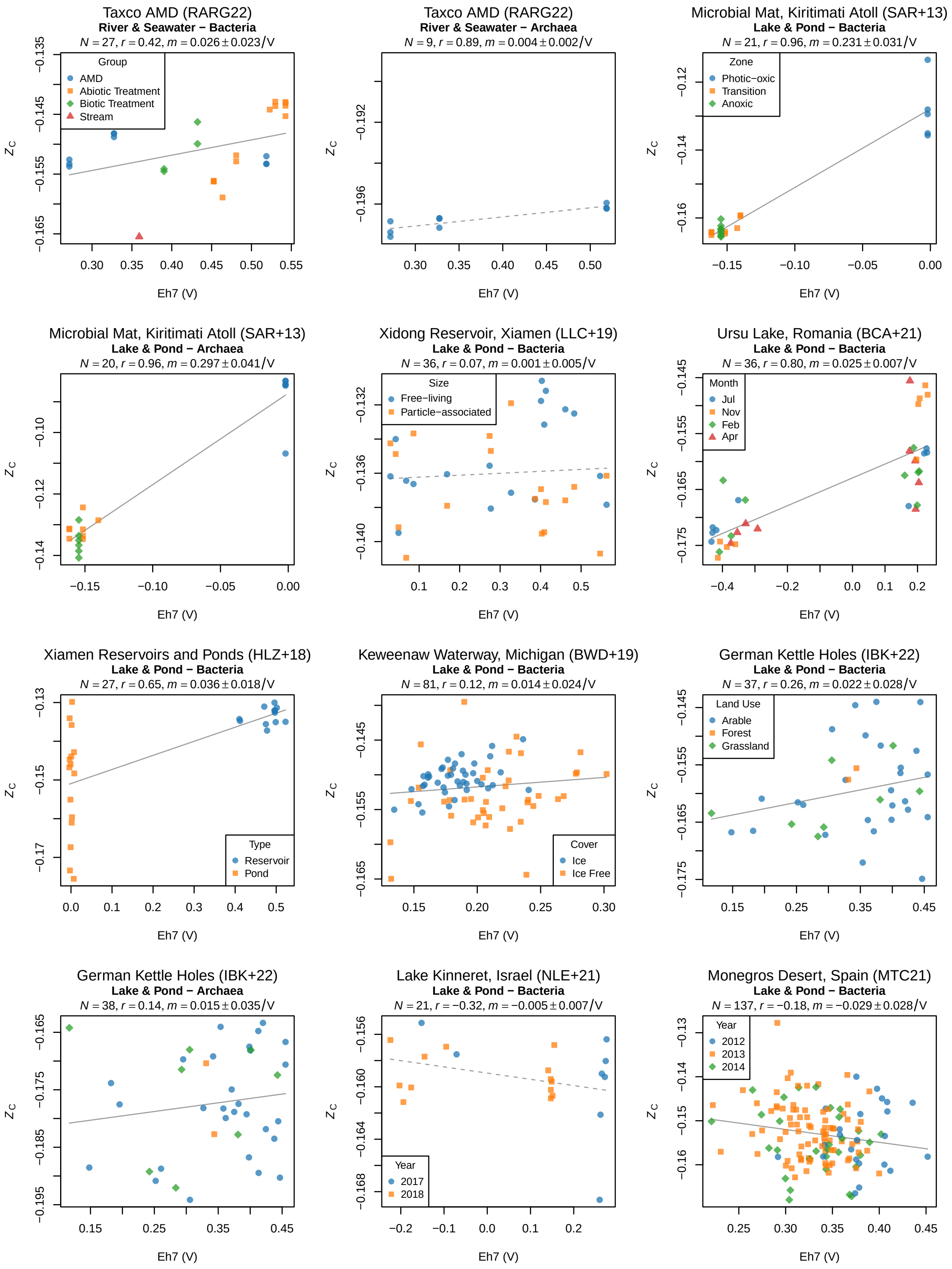

Figure S1. (Continued) AMD – acid mine drainage.

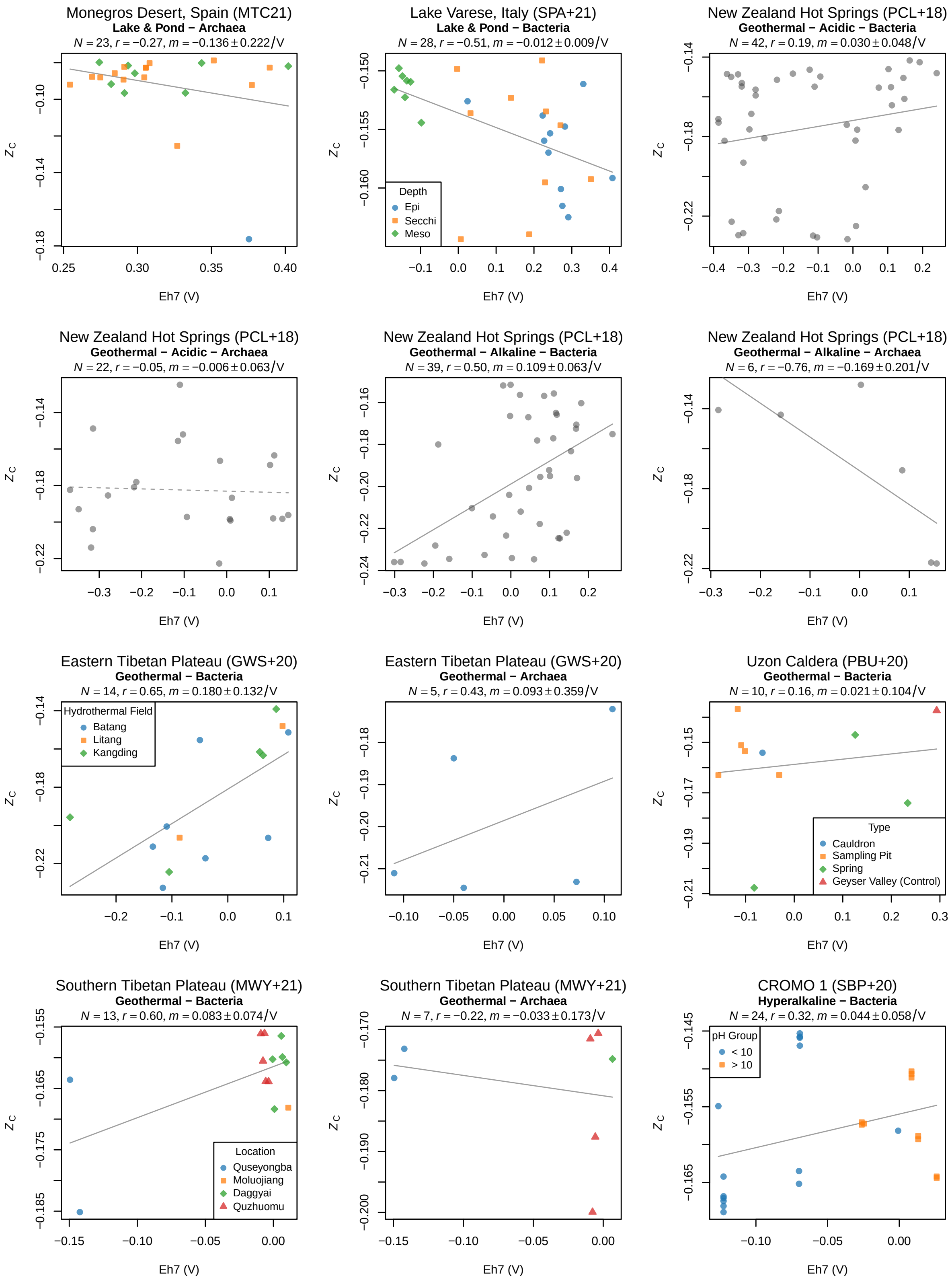

Figure S1. (Continued) Epi – epilimnion; Secchi – 2.5 × the Secchi depth; Meso – mesolimnion; CROMO – Coast Range Ophiolite Microbial Observatory.

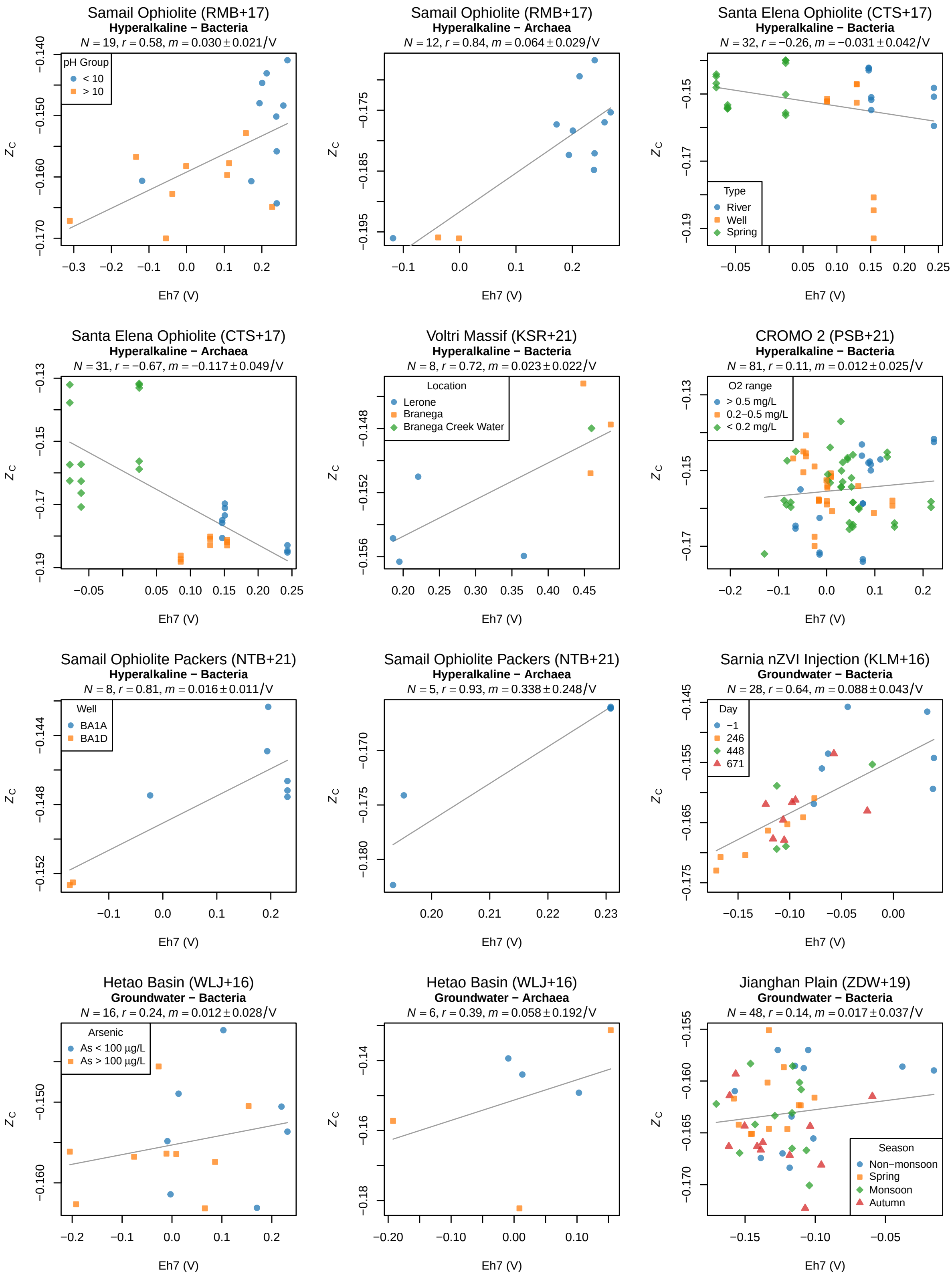

Figure S1. (Continued) nZVI – nanoscale zero-valent iron.

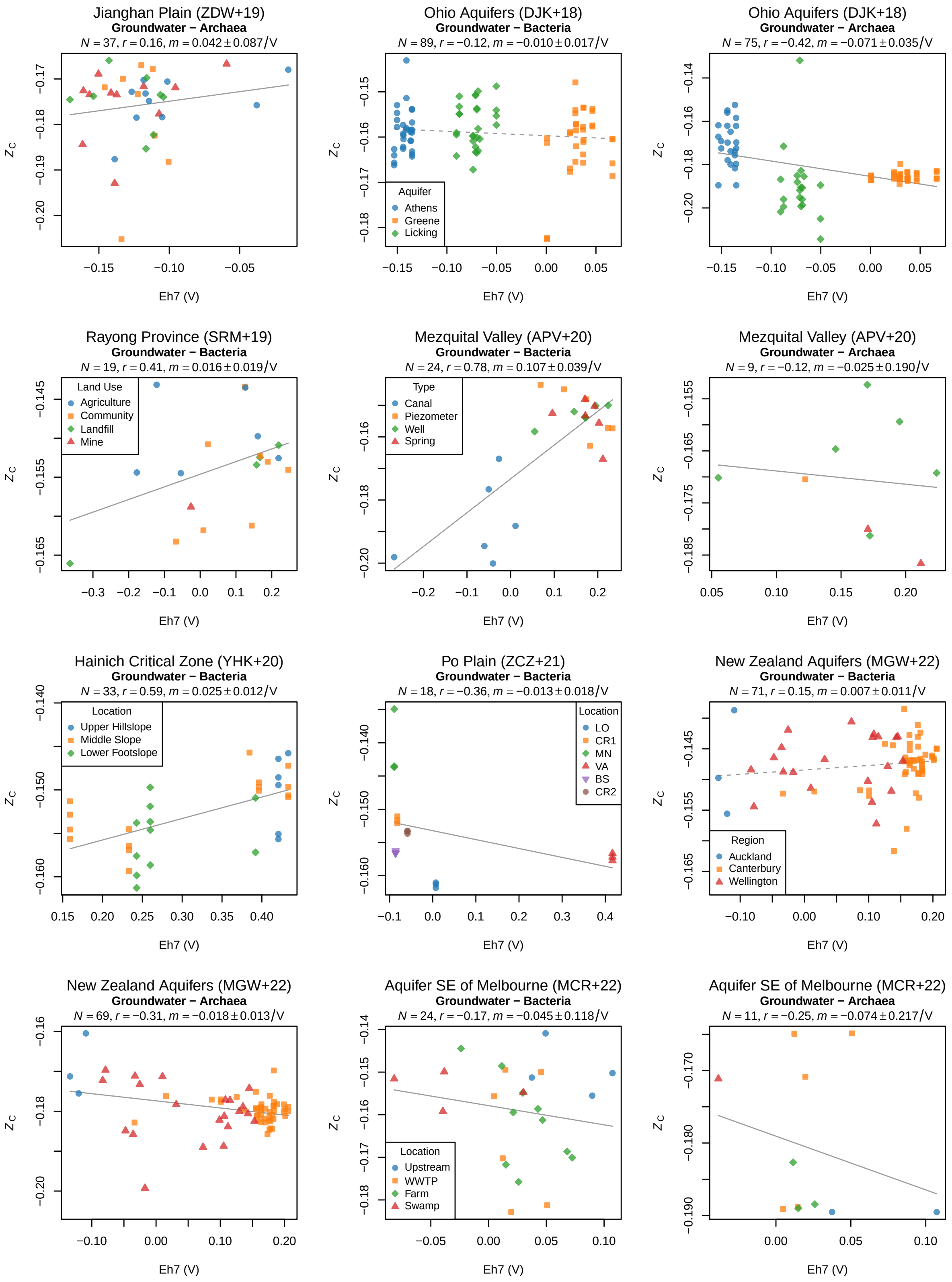

Figure S1. (Continued) VA, LO, CR1, CR2, BS, MN – Provinces of Varese, Lodi, Cremona, Brescia, and Mantova in Northern Italy.

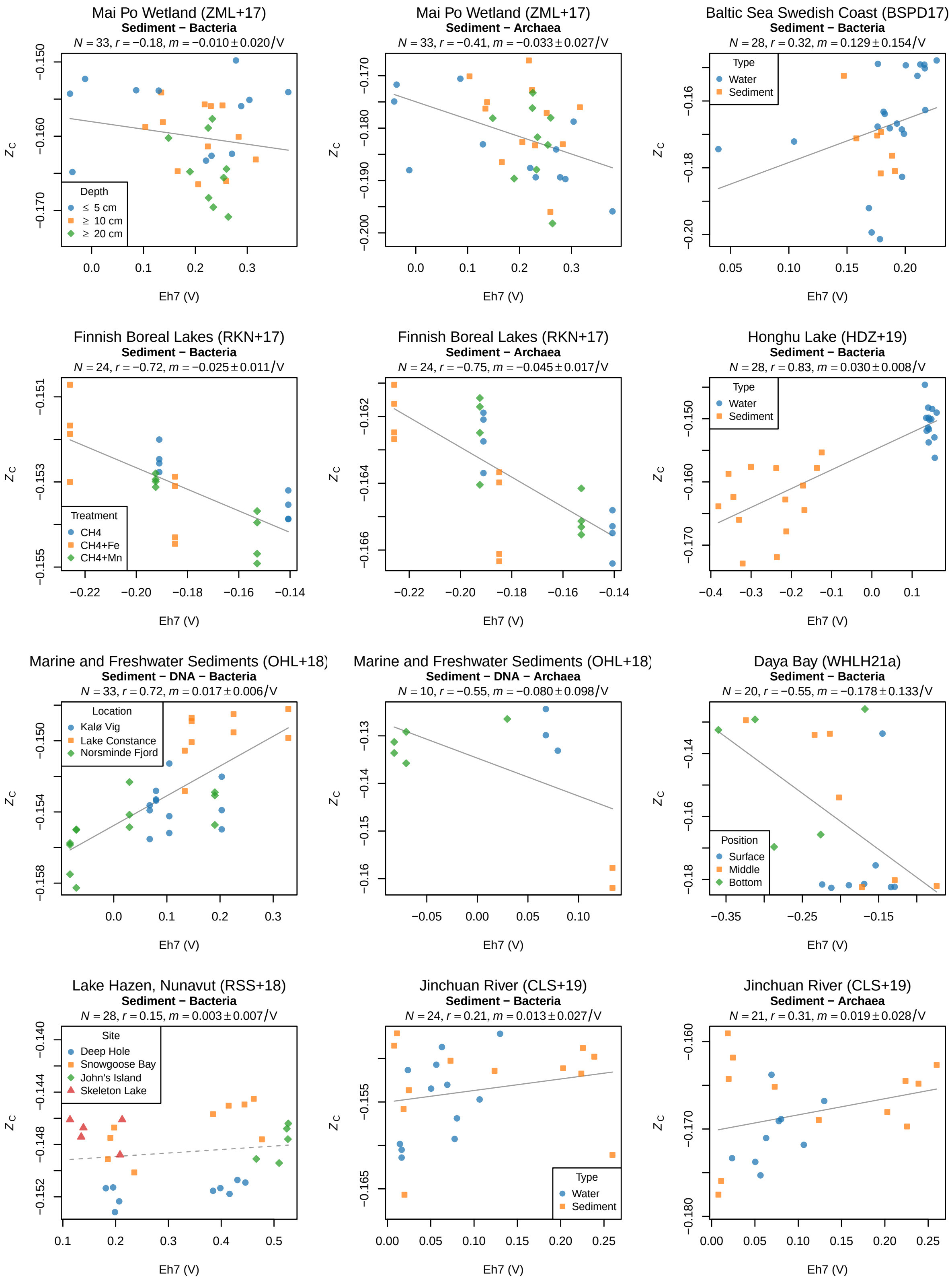

Figure S1. (Continued)

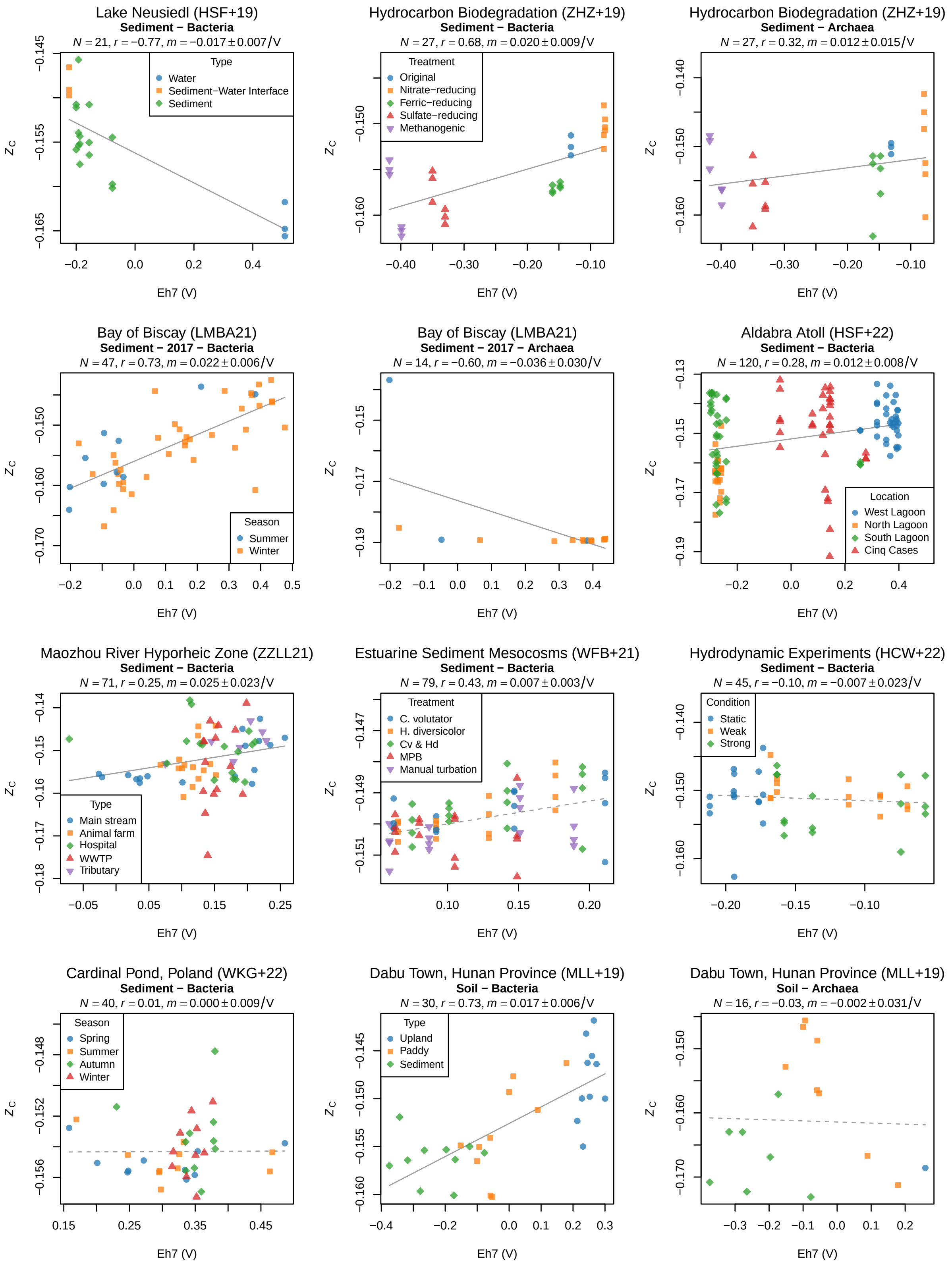

Figure S1. (Continued) Cv – *Corophium volutator*; Hd – *Hediste diversicolor*; MPB – microphytobenthos.

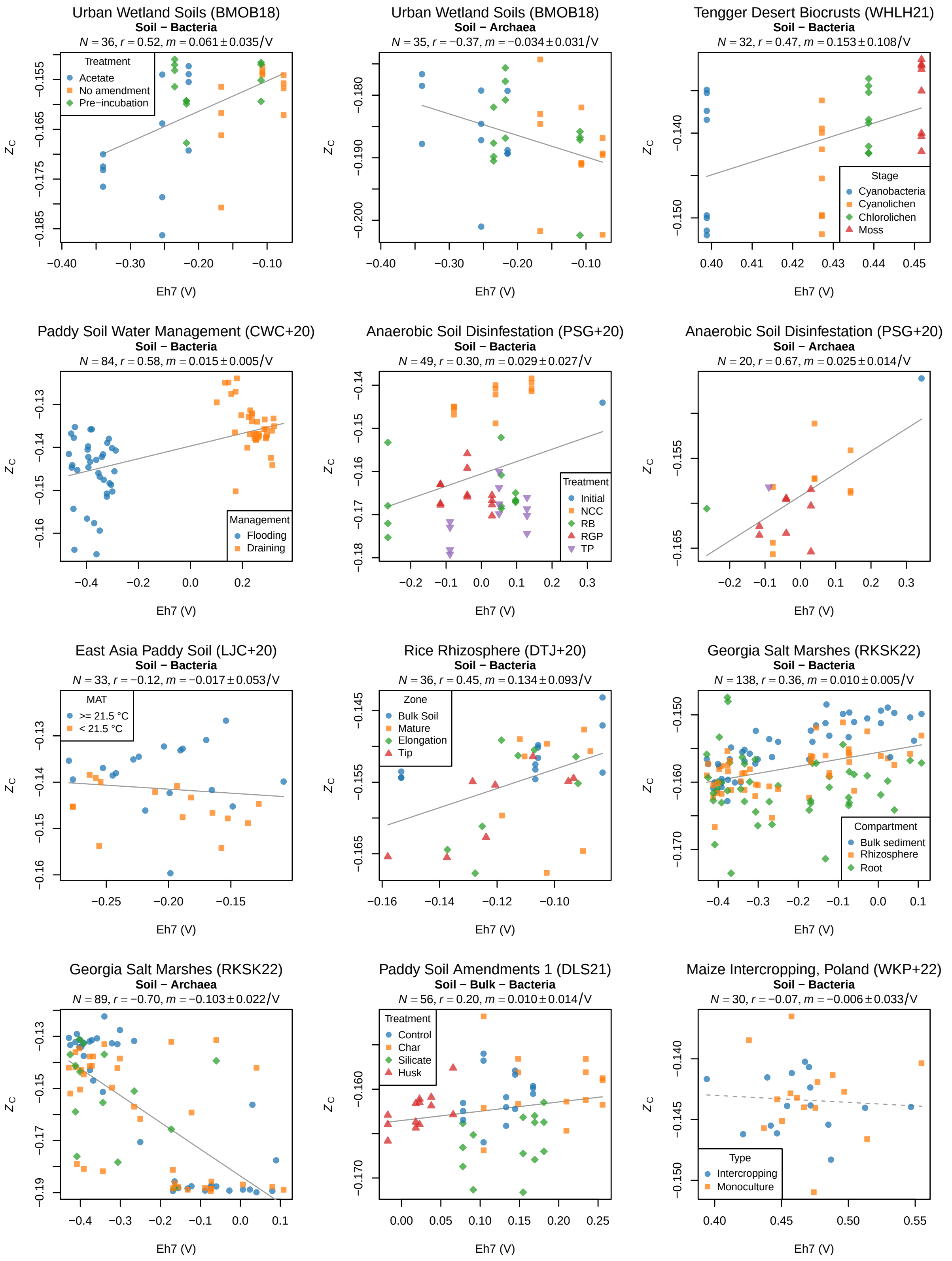

Figure S1. (Continued) NCC – no carbon control; RB – rice bran; RGP – red grape pomace; TP – tomato pomace; MAT – mean annual temperature.

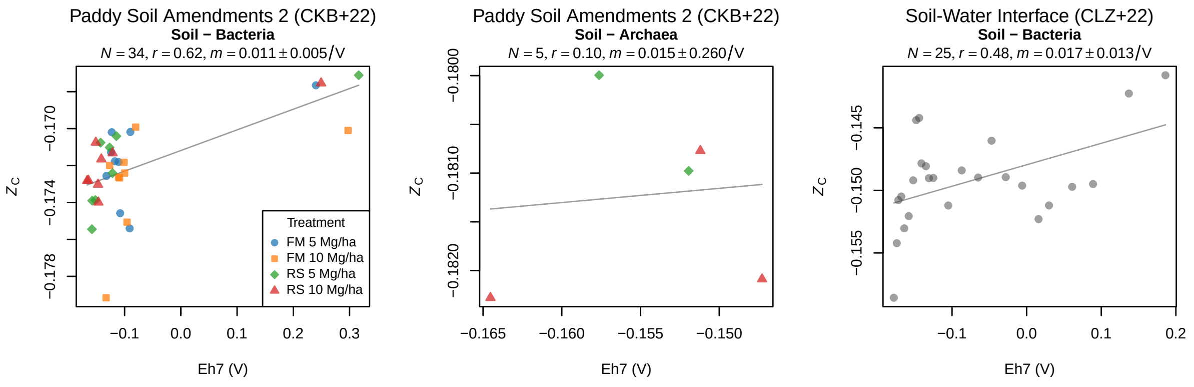

Figure S1. (Continued) FM – farmyard manure; RS – rice straw.
