# Supplementary material for: Community- and genome-based evidence for a shaping influence of redox potential on bacterial protein evolution
Source: mSystems. 2023 Jun 8;8(3):e00014-23. doi: 10.1128/msystems.00014-23 (PMC10308962; doi:10.1128/msystems.00014-23)
Supplement: FIG S2 — Comparison of Eh7, Eh, and O2 concentration as predictors of carbon oxidation state. Within each domain, all plots represent the same set of samples (i.e., those for which O2 measurements are available). The number of data sets is indicated by bold numbers at the top left of each plot; the number of samples and slope of the linear regression ± the margin of error for the 95% confidence interval are shown at upper right; the Pearson correlation coefficient is shown at bottom right. The numbers of samples for each environment type are listed in the bottom legends. [file msystems.00014-23-s0002.pdf]

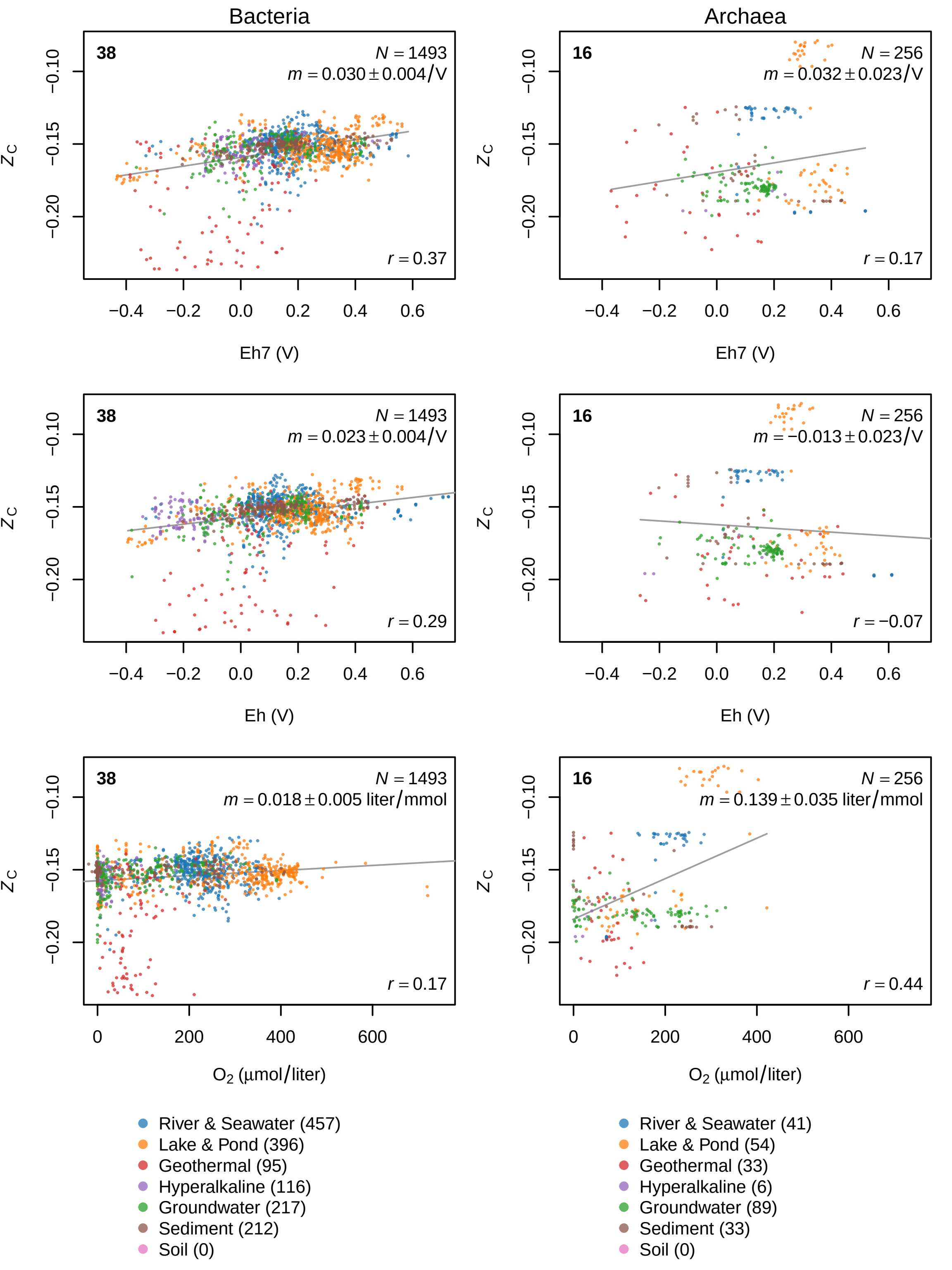

Bacteria
Archaea
38
N = 1493
m = 0.030 ± 0.004/V
r = 0.37
16
N = 256
m = 0.032 ± 0.023/V
r = 0.17
Eh7 (V)
38
N = 1493
m = 0.023 ± 0.004/V
r = 0.29
16
N = 256
m = −0.013 ± 0.023/V
r = −0.07
Eh (V)
38
N = 1493
m = 0.018 ± 0.005 liter/mmol
r = 0.17
16
N = 256
m = 0.139 ± 0.035 liter/mmol
r = 0.44
O2 (µmol/liter)
ZC
River & Seawater (457)
Lake & Pond (396)
Geothermal (95)
Hyperalkaline (116)
Groundwater (217)
Sediment (212)
Soil (0)
River & Seawater (41)
Lake & Pond (54)
Geothermal (33)
Hyperalkaline (6)
Groundwater (89)
Sediment (33)
Soil (0)
