# Supplementary figures and images for: Community- and genome-based evidence for a shaping influence of redox potential on bacterial protein evolution
Source: mSystems. 2023 Jun 8;8(3):e00014-23. doi: 10.1128/msystems.00014-23 (PMC10308962; doi:10.1128/msystems.00014-23)

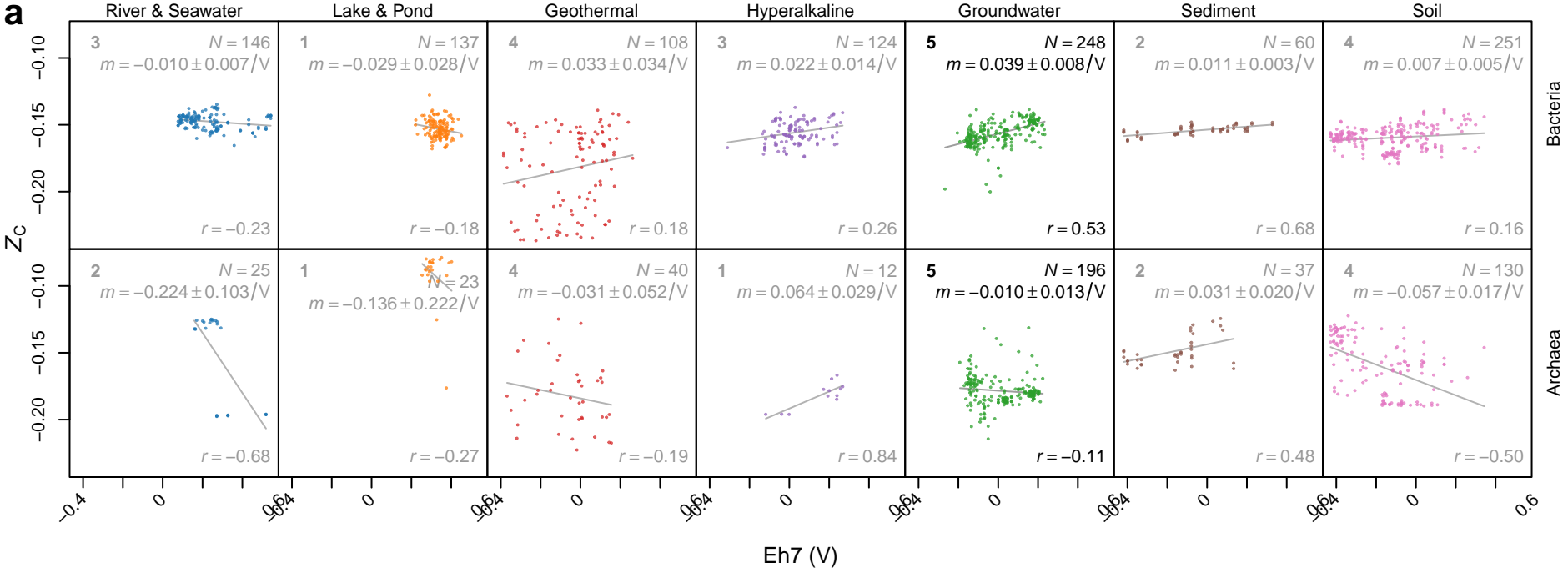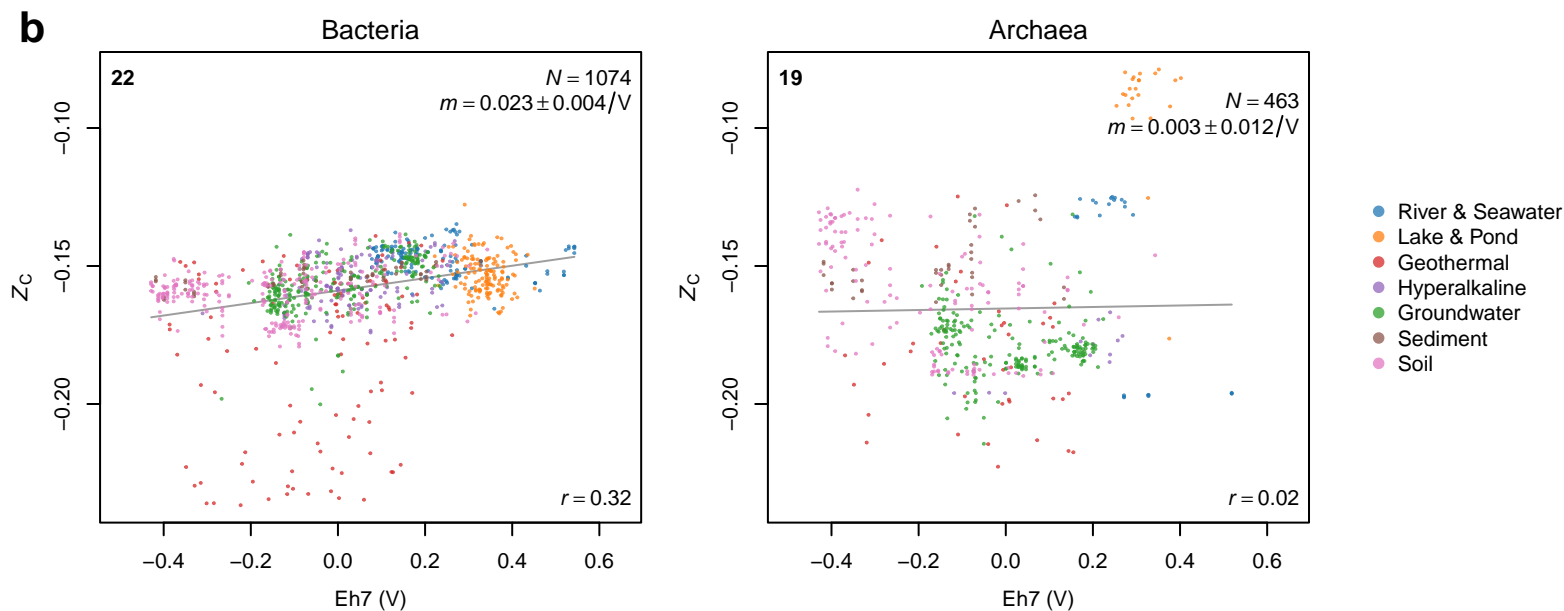

Supplement: FIG S3 — Global analysis including only data sets generated with 515F/806R primers (these data sets are identified in Table S1). This figure was made analogously to Fig. 6 [file msystems.00014-23-s0003.pdf]
